# Beyond traditional coconut oil: metabolic effects of medium-chain triglyceride oil-mechanistic insights, lipid remodeling, and clinical implications in cardiometabolic health

**DOI:** 10.3389/fphys.2026.1966609

**Published:** 2026-10-13

**Authors:** Reham M. Algheshairy

**Affiliations:** Department of Food Science and Human Nutrition, College of Agriculture and Food, Qassim University, Buraydah, Saudi Arabia

**Keywords:** cardiometabolic health, coconut oil, ketogenesis, lipid metabolism, medium-chain triglycerides, mitochondrial oxidation

## Abstract

Medium-chain triglyceride (MCT) oil and traditional coconut oil are frequently regarded as comparable lipid sources; however, their metabolic effects differ considerably due to variations in fatty acid composition, absorption pathways, and biological processing. Coconut oil contains substantial amounts of lauric acid and long chain saturated fatty acids (LCSFAs), whereas purified MCT oils are enriched with caprylic (C_8_) and capric (C_10_) fatty acids, which demonstrate distinct metabolic behavior. This review provides a comprehensive analysis of the mechanistic basis underlying MCT-mediated metabolic remodeling, with emphasis on lipid metabolism, mitochondrial function, circulating lipid regulation, and cardiometabolic implications. MCTs facilitate preferential oxidative metabolism through rapid portal absorption, enhanced mitochondrial β-oxidation, ketogenesis, and thermogenic responses. Furthermore, MCT-derived metabolic signaling influences key regulatory pathways, including activation of peroxisome proliferator-activated receptors (*PPARs*), suppression of sterol regulatory element-binding protein-1c (*SREBP-1c*)-mediated lipogenesis, and modulation of the gut microbiota-lipid metabolism-inflammation axis. These mechanisms contribute to improved metabolic flexibility, altered lipid partitioning, and potential benefits in dyslipidemia, obesity, and insulin resistance. However, clinical outcomes remain dependent on formulation characteristics, dosage, duration of intervention, and individual metabolic status. Distinguishing purified MCT oil from conventional coconut oil is essential for accurate interpretation of lipid research and for developing targeted nutritional strategies aimed at improving cardiometabolic health.

## Graphical Abstract

**Figure:**
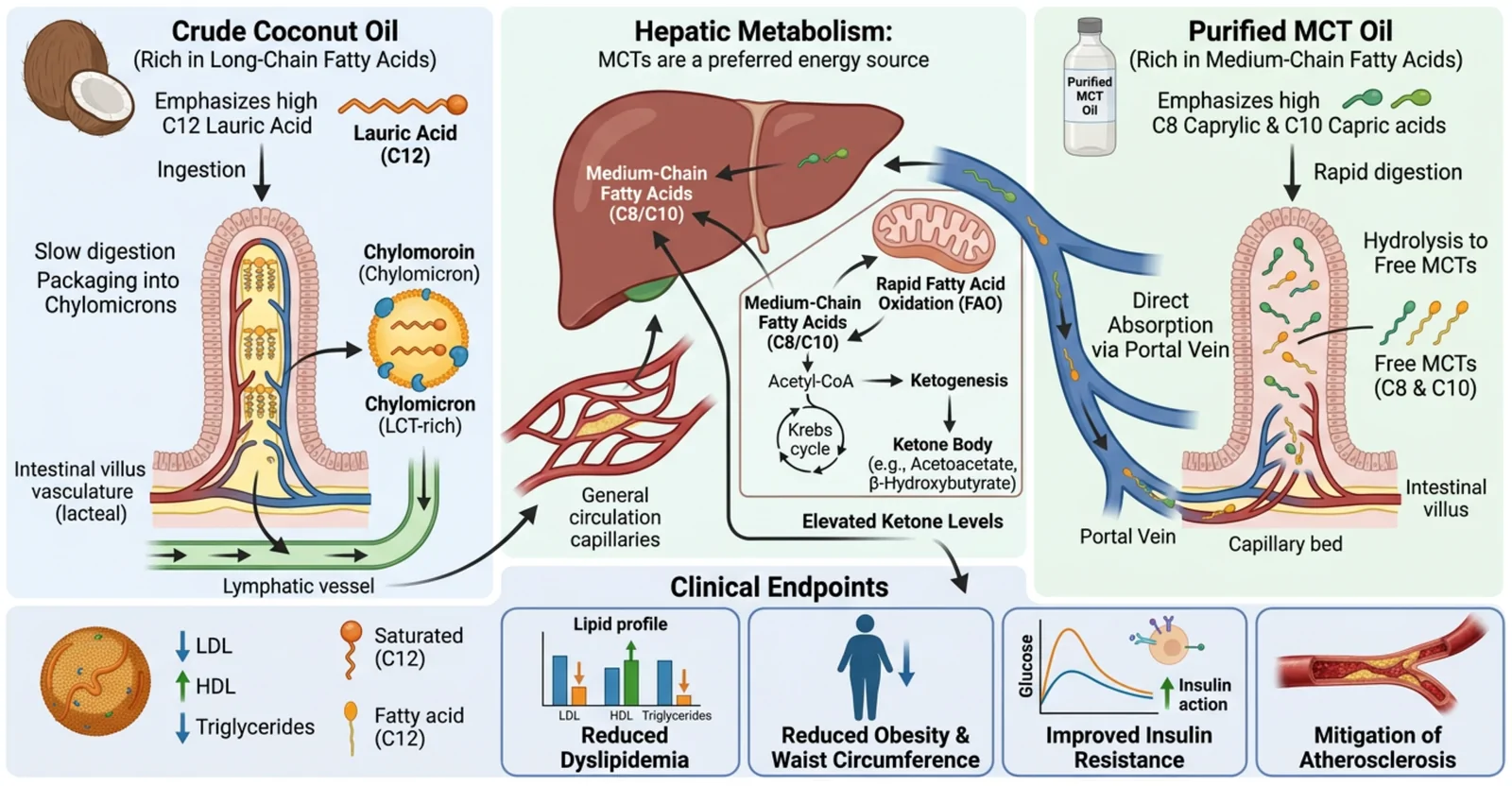

## 1 Introduction

The current global context of lipid nutrition is characterized by a strong lipid nutrition paradox with a focus on the intake of raw coconut oil and its metabolic consequences for cardiometabolic health. Coconut oil is traditionally linked to increased atherosclerotic risk in Western epidemiological lipid research cohorts, often because of its saturated fatty acid (SFA) content, which is typically more than 90% in the majority of oil sources, and may lead to increased low-density lipoprotein cholesterol (LDL-C) (Sacks et al., 2017; Neelakantan et al., 2020). Longitudinal studies reveal that high consumption of coconut lipids is associated with a low prevalence of cardiovascular disease and a favorable cardiovascular risk profile in indigenous people in Pukapuka and Tokelau (Teng et al., 2020). This is mainly because of the fundamental chemical classification difference between the saturated lipid subclasses, in this case the difference between long-chain triglycerides (LCTs, >C14) and the medium-chain triglycerides (MCTs, C6-C12) (Dayrit, 2015). MCTs and LCTs differ fundamentally in digestion, transport and oxidative fate (Jadhav and Annapure, 2023; Augustin et al., 2018). The industrial fractionation of lipids has led to a key difference between unrefined coconut oil, which is a mixture of medium-chain fatty acids (MCFAs) and long-chain triglycerides (LCTs), with approximately 50% being the lauric acid (C12:0) form, and pharmaceutical-grade purified MCT oil, standardized to be C8/C10 dominant (Duranova et al., 2025). Lauric acid is an intermediate-chain fatty acid (ICFA), but has physiologic characteristics of LCTs: more dependent on lymphatic transport and slower rate of hepatic oxidation than C8 and C10 (Norgren et al., 2020). This biochemical nuance has led to a persistent translational confusion in lipid research, where clinical literature frequently conflates the bioactivity of whole coconut oil with that of purified MCT lipid formulations. This amalgamation leads to an inaccurate assessment of the hypercholesterolemic activity of the lauric and myristic acid sub-components of whole oil and is frequently assigned to the entire MCT class (Deen et al., 2021). Thus, studies combining coconut oil interventions with purified MCT supplements tend to yield contradictory results for the effect of coconut oil on LDL-C and apolipoprotein B (ApoB), which are unique regulatory pathways of isolated C8/C10 fractions (Kinsella et al., 2017). Current lipid reviews often do not distinguish MCT-specific lipid regulatory pathways from the noxious lipid signals of lauric acid and LCT that are already present in whole coconut oil (Schwingshackl and Schlesinger, 2023). The presence of lauric acid in study diets can also confound the precise metabolic effect of the shorter-chain MCTs on circulating lipid remodeling because of its ability to raise total serum cholesterol levels and, at the same time, raise high-density lipoprotein (HDL) levels (Chatturong et al., 2023). There are differences in the sensitivity to MCT-induced ketogenesis and lipid clearance in dyslipidemic populations, in metabolic syndrome and different age cohorts (Abe et al., 2016). Also, there is no apparent formulation-specific dose-lipid response framework to inform on lipid-specific nutritional intervention, as most clinical recommendations are currently lacking in precision to distinguish the effects of different C8:C10 ratios or even specific dosages on the lipidome (Abe et al., 2016). In addition, the lipid risk/benefit profile of the different levels of energy expenditure and insulin sensitivity is not well defined, and this makes the development of therapeutic lipid strategies for metabolic and cardiovascular disorders difficult (Titov et al., 2019). The primary objective of this review is to address these deficiencies by dissecting pure MCT lipid metabolic mechanisms independent of coconut oil LCT interference via direct lipid phenotype comparison. By analyzing the influence of formulation ratios, dosage, duration, and the metabolic status of study populations, we seek to resolve cross-study lipid response discrepancies that have historically hindered clinical consensus. By clearly distinguishing the culinary utility of coconut oil from the targeted therapeutic application of purified MCT oils, we provide a rigorous framework for managing health and lipid-related diseases in the modern clinical landscape.

## 2 Structural and physiological lipid dichotomy: traditional coconut oil vs. purified MCT oil

### 2.1 Fatty acid fingerprint and lipid formulation heterogeneity

Crude coconut oil (CCO) has a distinct biochemical architecture, a “lipid fingerprint”, which is comprised of a heterogeneous mixture of fatty acids that cannot be easily categorized by metabolism. Although it is often marketed as a rich source of MCTs, the lipid profile of coconut oil is composed mainly of lauric acid (C_12_), which makes up around 48-52% of the total lipid profile (Chatturong et al., 2023). Physiologically, lauric acid falls between the medium and long-chain fatty acids (LCFAs). Unlike true MCTs, most lauric acid (about 70–75%) is absorbed through chylomicrons and is carried in the lymphatic system instead of the portal vein (McCarty and DiNicolantonio, 2016). This structural ambiguity makes it a pro-atherogenic source of lipids, with an elevation of low-density lipoprotein (LDL) cholesterol levels, but also gives it a mild metabolic benefit by dramatically raising high-density lipoprotein (HDL) cholesterol levels, which can lead to a neutral or favorable TC-to-HDL ratio. This MCFA-LCT hybrid metabolism inherently limits the physiological effect of coconut oil, as it does not produce the rapid hepatic oxidation and acute ketosis noted when using a purified form of coconut oil. In contrast, purified MCT oil is a standardized lipid formulation engineered to eliminate the long-chain lipid contamination and metabolic lag associated with C_12_. These oils are normally produced by molecular distillation of coconut or palm kernel oil, where caprylic acid (C_8_) and capric acid (C_10_) are concentrated and lauric acid (C_12_) is minimized to a very low level (Wajs and Brodziak, 2022).

These lipids are not transported via the chylomicron pathway in the lymphatics, and so they are rapidly taken up into the portal circulation and transported directly to the liver for β-oxidation. The streamlined physiology makes purified MCTs a powerful and non-carbohydrate energy source. This fingerprint is recently shown to be very effective in modulating metabolic markers, compared with coconut oil, and has been demonstrated to be more effective in reducing ectopic fat deposition and improving energy expenditure (Kinsella et al., 2017). Commercial subtyping of MCT oils further elicits these structure–lipid function relationships by utilizing specific ratios of C_8_ to C_10_. Ketogenic formulations containing C_8_-predominant (caprylic acid) are the true “gold standard” in terms of the ketogenic effect, and there is a direct linear relationship between plasma ketone levels and the rapid hepatic oxidation. C_10_-predominant (capric acid) formulations are metabolically superior than LCTs, but have a slightly slower oxidation rate and a lower ketogenic ceiling than C_8_ (Vandenberghe et al., 2017). The balanced C_8_/C_10_ mixtures have proven to be very effective clinically, balancing weight management and satiety signaling. These balanced blends have been shown to be more effective at reducing hunger hormones and reducing adiposity when fed to animals on a high fat diet, in part due to the lack of metabolic “dilution” because of the C_12_-LCT portion of conventional oils (Wali et al., 2020). So the difference between traditional coconut oil and purified MCTs is not simply a purity issue, but also a fundamental difference in how the oil is metabolized in the body and its effectiveness. Fatty acid composition of CCO and commercial purified MCT oil formulations is summarized in (**Table 1**).

**Table 1:** Fatty acid composition, medium-chain fatty acid content and typical industrial application of crude coconut oil and commercial purified MCT oil formulations.

Fatty acid	Carbon chain	Crude coconut oil (%)	Purified MCT oil, C8:C10 blend (%)	Purified C8 oil, tricaprylin (%)	Reference
Caproic acid	C6:0	0.4-0.8	0.5-2.0	<0.5	(Dale and Meara, 1955; McCarty and DiNicolantonio, 2016)
Caprylic acid	C8:0	5.0-9.0	50.0-70.0	>95.0	(Kanprakobkit et al., 2023; Vandenberghe et al., 2017)
Capric acid	C10:0	5.0-10.0	30.0-50.0	<4.0	(Ghosh and Bhattacharyya, 1997; Narwal et al., 2025)
Lauric acid	C12:0	45.0-52.0	<2.0	<1.0	(Arpi, 2014; Chatturong et al., 2023)
Myristic acid	C14:0	16.0-21.0	Trace	ND	(Arpi, 2014)
Palmitic acid	C16:0	7.0-10.0	ND	ND	(Arpi, 2014)
Stearic acid	C18:0	2.0-4.0	ND	ND	(Dale and Meara, 1955)
Oleic acid	C18:1	5.0-8.0	ND	ND	(Dale and Meara, 1955)
Linoleic acid	C18:2	1.0-3.0	ND	ND	(Dale and Meara, 1955)
Total MCFA (C6:0-C12:0)	–	~63.5	~100	~100	(Dale and Meara, 1955; Narwal et al., 2025)
Total C14-C18 LCFA	-	35.0-40.0	<1.0	Trace	(Arpi, 2014; Narwal et al., 2025)
Typical industrial application	–	Food processing, cosmetics, oleogel synthesis	Medical nutrition (enteral/parenteral), ketogenic supplements	Pharmaceutical carrier, high-potency neuro-nutraceuticals	(Narwal et al., 2025; Vandenberghe et al., 2017)

ND, not detected or present only in negligible trace amounts; - = not applicable. Percentages represent weight percent (wt.%) of total fatty acids.

### 2.2 Post-ingestive lipid processing and systemic lipid trafficking

The post-ingestive processing of dietary lipids is governed by a fundamental structural dichotomy between LCTs and MCTs, which dictates their subsequent systemic trafficking and metabolic fate (**Figure 1**). Traditional LCTs are prevalent in the dietary fats used in traditional coconut oil, and require a multi-stage digestion process that starts with emulsification in the small intestine by bile salts, followed by hydrolysis by pancreatic lipase (Cheng et al., 2024). Upon absorption, the LCFAs are then obligately re-esterified to TGs in enterocytes, which are subsequently packaged into large chylomicrons. These lipoproteins are unable to cross the capillary, but are secreted into the lymphatic system through the lacteals, thus avoiding first-pass hepatic metabolism (Cheng et al., 2024). LCT-derived lipids are transported systemically through the lymphatic circulation to peripheral tissues, where they are mainly deposited in lipid compartments for storage in white adipose tissue, or under conditions of caloric excess, pathological ectopic deposition in organs other than adipose tissue, including the liver and skeletal muscle. In contrast, purified MCT oil takes a ‘privileged’ metabolic route that is rapidly metabolized by both absorption and oxidation. MCTs are hydrolyzed largely independently of bile salts and can be broken down efficiently, even in the presence of biliary insufficiency, as a result of their smaller molecular size and greater water solubility (Cao et al., 2025). The resulting MCFAs are not re-esterified into chylomicrons, but are absorbed directly as free fatty acids (FFA) to the portal venous system. This direct pathway allows direct delivery of MCFAs to the liver, where they are rapidly metabolized by mitochondrial β-oxidation (Lee et al., 2022). In contrast to LCFAs which must rely on the carnitine palmitoyltransferase-1 (CPT-1) shuttle to gain access to the mitochondria, MCFAs (especially C8 and C10) can enter the mitochondria without the need for the rate limiting CPT-1 shuttle. This allows for rapid oxidation and generation of ketone bodies, a readily used systemic energy source, especially for the brain and cardiac tissue. Therefore, the MCT pathway is associated with low storage of lipids, because the liver favors their oxidation over esterification into very-low-density lipoproteins (VLDL) (Taylor et al., 2019). The systemic effects of these two classes of TGs on whole body lipid homeostasis are quite distinct. The LCT lymphatic pathway is energy storing and long duration, which correlates with an expansion of adipose stores. In contrast, the high-activity lipid turnover and thermogenesis of the oxidation prone MCT portal pathway may help to increase satiety and decrease the cumulative risk of metabolic dysregulation (Cao et al., 2025). This metabolic shift makes purified MCT oil more clinically useful than conventional fats in applications such as malabsorption disorders, neurodegenerative diseases, and others that demand fast mobilization of energy sources other than glucose (Taylor et al., 2019).

**Figure 1:**
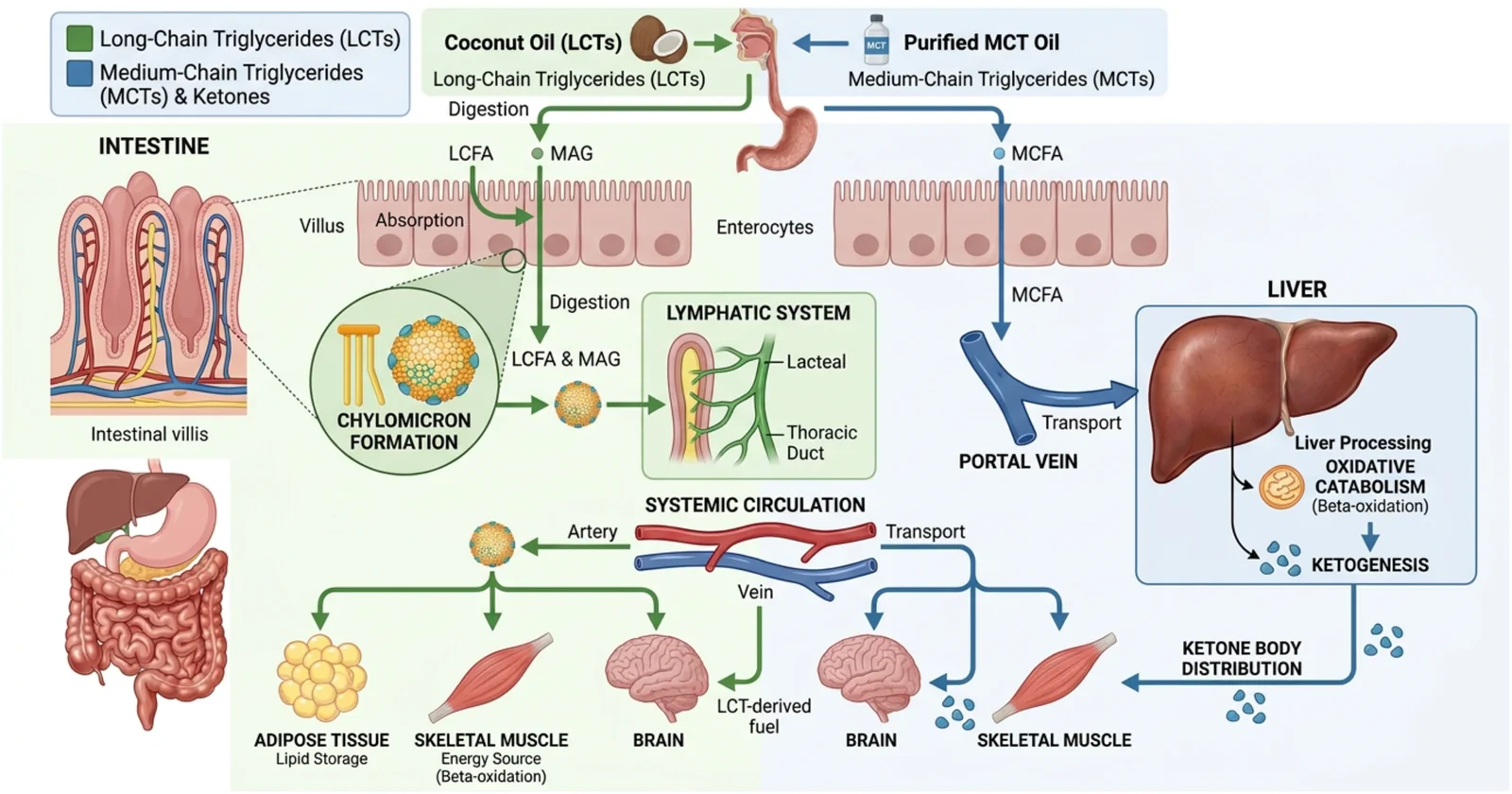
Schematic comparison of intestinal digestion, absorption and systemic lipid trafficking of LCTs (Coconut Oil) vs MCTs.

### 2.3 Intrinsic lipid metabolic superiority of refined MCT lipids

The metabolic advantages of refined MCT lipids over conventional LCTs and CCO are mainly linked to the specific structure and subsequent metabolism of MCT lipids. Refined MCTs possess fatty acid chains of 6–12 carbons and have demonstrated increased systemic lipid flexibility by shifting lipid metabolism towards oxidative catabolism and away from *de novo* lipogenic storage (Cao et al., 2025). This shift follows directly from the trafficking differences as described above (Lima et al., 2021). Because of this, refined MCTs are more likely to be used for energy generation than storage, which diminishes the incorporation into peripheral adipose tissue or ectopic deposition in the liver and skeletal muscle (McCarty and DiNicolantonio, 2016). This oxidative bias has been shown to inhibit *de novo* lipogenesis by reducing the expression of important enzymes involved in it, which helps to reduce the formation of lipid metabolites that contribute to metabolic syndrome and insulin resistance (Watanabe and Tsujino, 2022). This transition helps to promote better thermogenesis as well as improved metabolic flexibility, which is the ability of the body to switch between different pathways of substrate oxidation to keep energy balance in check in response to fluctuating dietary intakes (Lee et al., 2022). Additionally, refined MCTs exhibit higher lipogenic plasticity in terms of their capacity to create a rapid, continuous supply of plasma ketone bodies β-hydroxybutyrate (βHB) and acetoacetate in normocaloric conditions (Vandenberghe et al., 2017). This nutritional ketosis is not induced by a low-carb diet or fasting, but rather by the surging amount of MCFAs that enter the hepatic mitochondrion when they exceed the capacity of the tricarboxylic acid cycle (TCA) cycle, forcing excess acetyl-CoA into ketogenesis. Ketogenic effectiveness is highly dependent on the carbon chain length; purified tricaprylin (C8) has been demonstrated to have a much greater acute ketogenic effect compared to the more gradual oxidation of traditional coconut oil that contains a high proportion of lauric acid (C12) (Cunnane et al., 2016). In disease states such as neurodegenerative disorders and cognitive impairment, where glucose metabolism is impaired, the continuous production of ketones from MCTs can help sustain cerebral bioenergetics without the interference of impaired glucose transporters (Taylor et al., 2019).

## 3 Mechanistic lipid signaling underlying MCT-driven metabolic remodeling

### 3.1 Mitochondrial lipid energetics and thermogenic lipid reprogramming

The switch from LCTs to MCTs triggers a profound metabolic shift of increased mitochondrial efficiency and activation of thermogenic pathways. The primary reason for this metabolic remodeling is due to the unique pharmacokinetic properties of MCFAs which are able to enter the mitochondrial matrix without the need for the CPT-1 transport system. This promotes quick β-oxidation and initiates a cascade of intracellular signaling events that promote energy expenditure over energy storage in lipids (Rial et al., 2020). One of the key mechanisms in this MCT-driven energetic reprogramming is the marked upregulation of mitochondrial uncoupling proteins (UCPs), including UCP1, UCP2, and UCP3. MCT supplementation has been shown to induce the expression of UCP1 in BAT and the beiging of WAT, which may be mediated through the activation of the adenosine monophosphate-activated protein kinase (AMPK) signaling pathway (Jia et al., 2025). MCTs increase the activity of the mitochondrial respiratory chain and uncoupling, thereby releasing the proton gradient across the inner mitochondrial membrane from ATP formation into the heat. MCFAs are ligands for PPARs transcriptionally regulating genes involved in mitochondrial biogenesis and oxidative capacity (Xia et al., 2022). Additionally, MCTs have a substrate-specific ability to increase postprandial lipid oxidation. The rapid portal influx of MCFAs triggers a burst of mitochondrial β-oxidation, leading to a significant increase in oxygen consumption and increased production of ketone bodies (McCarty and DiNicolantonio, 2016). Thermogenic response to MCT-enriched diets has been reported in many individuals, who show more total energy expenditure than those on isocaloric LCT diets, maybe due to the increased activity of the sympathetic nervous system and secretion of neurotrophic factors (Zulkanain et al., 2020). In addition to systemic thermogenesis, MCTs have a protective effect on ectopic lipid accumulation in non-adipose tissues, such as the liver and skeletal muscle, through modulation of molecular sensors of lipid status. This inhibition of lipid storage occurs via the co-suppression of important lipogenic regulators such as sterol regulatory element-binding protein 1c (SREBP-1c), fatty acid synthase (FAS) and acetyl-CoA carboxylase (ACC). At the same time, MCTs seem to influence mitochondrial lipid sensing through the decreased expression of CD36 (fatty acid translocase) and other transporters that promote the accrual of excess lipids in ectopic sites. This selective partitioning to catabolic pathways instead of intracellular storage is a key mechanism to consider when using MCTs for metabolic syndrome and associated comorbidities (Lee et al., 2018).

### 3.2 Ketogenic lipid signaling axis and glucose-lipid homeostasis crosstalk

Hepatic MCFA delivery through the portal vein ensures that the liver is immediately presented with a high flux of substrates for metabolic processing, and MCFAs enter the mitochondria without the rate-limiting CPT-1 shuttle, while LCFAs do require it (Cao et al., 2025). As a result, the hepatic mitochondrial pool quickly fills up with acetyl-CoA, more than can be utilized by the tricarboxylic acid (TCA) cycle, thus forcing an obligatory switch to ketogenesis. Recent data indicates that this strong production of ketone bodies, mainly β-hydroxybutyrate (βHB), is not only an alternative energy supply but a central metabolic remodeling mechanism via systemic lipid signaling (Kanta et al., 2025). βHB is a pleiotropic signaling metabolite that has effects via receptor-mediated mechanisms and direct enzyme modulation, and is a critical mediator of glucose-lipid homeostasis crosstalk. In particular, capric acid (C10) has been found to act as a ligand for the G-protein coupled receptor GPR40 (FFAR1) which regulates insulin secretion and increases the resistance of pancreatic β-cells to lipotoxic stress (Pujol et al., 2018). MCT-induced ketogenesis regulates the HCA2 (GPR109A) receptor that inhibits lipolysis in adipose tissue and limits the systemic pro-inflammatory state that frequently accompanies insulin resistance (Rial et al., 2020). This bi-directional regulation is supported by the fact that MCTs may reduce postprandial glucose concentrations and increase insulin sensitivity in peripheral tissues by increasing Akt phosphorylation (Abe, 2022). In addition to local hepatic effects, MCT promotes complex central-peripheral lipid crosstalk resulting in a more resilient metabolism in insulin-resistant dyslipidemic patients. Hepatic ketones are secreted to signal to skeletal muscle and adipose tissue, stimulating mitochondrial biogenesis and boosting oxidative metabolism through activation of PPAR-α (Aryal et al., 2025). One of the pivotal molecules in this crosstalk is the axis of CREBH-FGF21 signaling. MCFAs have been demonstrated to activate Cyclic AMP-responsive element-binding protein H (CREBH) and to promote the expression of fibroblast growth factor 21 (FGF21). This hormone-like protein exerts a systemic effect to suppress hepatic fat accumulation, induce thermogenesis and enhance lipid clearance, which thus lowers the markers of atherogenic dyslipidemia (Cao et al., 2024). In the context of metabolic syndrome, this signaling network promotes metabolic flexibility- the ability of the organism to efficiently switch between fuel sources. The sustained elevation of circulating ketones and MCFAs provides a protective buffer against the lipotoxicity inherent in high-fat diets, protecting distal organs from metabolic overload (Pujol et al., 2018).

### 3.3 Transcriptional lipid turnover control

The key mechanism for adiposity suppression by MCTs involves a potent inhibition of the SREBP-1c signaling pathway. SREBP-1c is the central transcriptional factor that regulates the expression of key enzymes involved in *de novo* lipogenesis (DNL) including FAS and ACC. MCFAs inhibit the proteolytic activation and nuclear translocation of SREBP-1c in the hepatic and adipose tissues (Jadhav and Annapure, 2023). This inhibition leads to a substantial decrease in the rate of DNL, preventing the assembly of TGs and their secretion in very low density lipoprotein (VLDL). MCTs inhibit the SREBP-1c-dependent lipogenic program and effectively uncouple the storage function of the liver from its metabolic energy (Yu et al., 2026). Conversely, MCTs are high affinity ligands for the PPAR family, inducing a catabolic switch through activation of *PPAR-γ* and *PPAR-α*. MCFAs activate *PPAR-α* in the liver to promote the transcription of genes that are critical for mitochondrial and peroxisomal β-oxidation including CPT1 and Acyl-CoA Oxidase. This upregulation will cause a rapid oxidation of the hepatic lipid pool, and prevent TGs from being formed. Moreover, under isocaloric substitution at moderate doses, MCFA-driven *PPAR-γ* activity in adipose tissue has a beneficial effect on lipid storage, increasing the production of adiponectin and the metabolism of fat stores (Watanabe and Tsujino, 2022). The direction of this *PPAR-γ* effect is not uniform, however, and depends on tissue, dose and prevailing energy balance; the apparently divergent findings obtained in isolated adipocytes and under caloric surplus. This *PPAR-α/γ* synergy will not only increase the catabolic flux of fatty acids but also promote remodeling of white adipose tissue into a more metabolically active beige-like phenotype, further enhancing energy expenditure (Ye et al., 2019). MCTs induce a state of metabolic resilience through both turning off the fat synthesis machinery and turning on the fat burning machinery, so preventing hypertriglyceridemia and hepatic steatosis (Yue et al., 2024; Cao et al., 2024). The *ChREBP* and the *HNF4-α* are ancillary factors in this MCT-controlled network that maintain glycemic and lipid homeostasis in response to nutritional excess (Lu, 2016). Thus, the transcriptional regulation from MCTs gives a strong molecular basis for their potential therapeutic use in dyslipidemia and metabolic syndrome, as compared to long-chain dietary fats (Heidt et al., 2026).

### 3.4 Gut microbiota-lipid metabolism–inflammation axis modulation

MCTs are not only fast-burning energy sources, but powerful biological modulators that interfere in a pathophysiological signaling pathway consistently impaired in dyslipidemia, obesity, and other chronic metabolic diseases: gut microbiota-lipid metabolism-inflammation. Longitudinal metabolic cohort studies have confirmed that MCT intervention increases gut microbial alpha-diversity which is known as a marker of intestinal homeostasis and host metabolic resilience (Zhang et al., 2022). In particular, MCT intake promotes an outgrowth of beneficial commensal genera such as Bifidobacterium and Turicibacter while medium-long-medium (MLM) structured TGs promote a selective enrichment of SCFA producing microbial taxa represented by Blautia and Anaerotruncus. Concurrently, MCT consumption inhibits growth of dysbiotic and pro-inflammatory pathogenic bacteria, including Ruminococcus torques and Eubacterium fissicatena group (Yue et al., 2020). This microbial reshaping further restores the Firmicutes/Bacteroidetes ratio, a microbial signature that is extensively altered, and often abnormally elevated, during high fat diet stress and obesity associated dyslipidemia (Shao et al., 2026). SCFA-producing commensal bacteria increase acetate, propionate and butyrate levels in the intestine, which supplies nutrients to colonocytes, strengthens the barrier function of the gut, and indirectly moderates lipid metabolism disturbance (Li et al., 2023). In addition to the SCFA-mediated epithelial protection, microbial alteration of endogenous bile acid pools regulates the activity of two key bile acid-sensing receptors, namely farnesoid X receptor (FXR) and G protein-coupled bile acid receptor 1 (GPBAR1/TGR5) (Zhao et al., 2022). Preliminary studies in preclinical models indicate that manipulation of bile acid metabolism with MCTs is effective in the treatment of non-alcoholic fatty liver disease (NAFLD) primarily by enhancing hepatic FXR signaling to coordinate systemic cholesterol homeostasis and *de novo* bile acid production, which results in lipid clearance (Yue et al., 2024). Apart from metabolic reprogramming, gut microbial optimization with MCTs also reduces metabolic endotoxemia and subsequent lipid-induced low grade metaflammation which is a second crucial protective mechanism against cardiometabolic decline. MCT supplementation preserves the intestinal mucosal morphological structure and increases the expression of epithelial tight-junction proteins to limit intestinal permeability and prevent portal translocation of gut-derived lipopolysaccharide (LPS) to systemic circulation. Limited endotoxin leakage additionally disrupts initial and further development of chronic metaflammation which is a pathological driver connected to dysregulated lipid metabolism and the progression of atherogenic dyslipidemia, and is linked to insulin resistance (Rial et al., 2016). In atherosclerosis animal models, anti-inflammatory phenotypes generated by MCT are associated with reduced expression of vascular pro-inflammatory mediators, such as vascular cell adhesion molecule-1 (VCAM-1), monocyte chemoattractant protein-1 (MCP-1) and macrophage infiltration marker cluster of differentiation 68 (CD68) (Yue et al., 2020). Furthermore, the microbial metabolites produced by MCT are anti-inflammatory and inhibitory of the activation of the NLRP3 inflammasome and inflammatory pathways mediated by NF-κB, which ultimately reduces the inflammatory burden throughout the body (Yue et al., 2020). The clinical benefits of these gut-modulated metabolic and inflammatory changes are seen as improved circulating lipid profiles (increased LDL/HDL ratio and decreased fasting and postprandial TGs) (He et al., 2024). In short, MCT-mediated modulation of the gut microbiota-lipid metabolism-inflammation axis establishes a solid mechanistic framework for the translation of MCTs to the management of dyslipidemia and metabolic syndrome (**Figure 2**).

**Figure 2:**
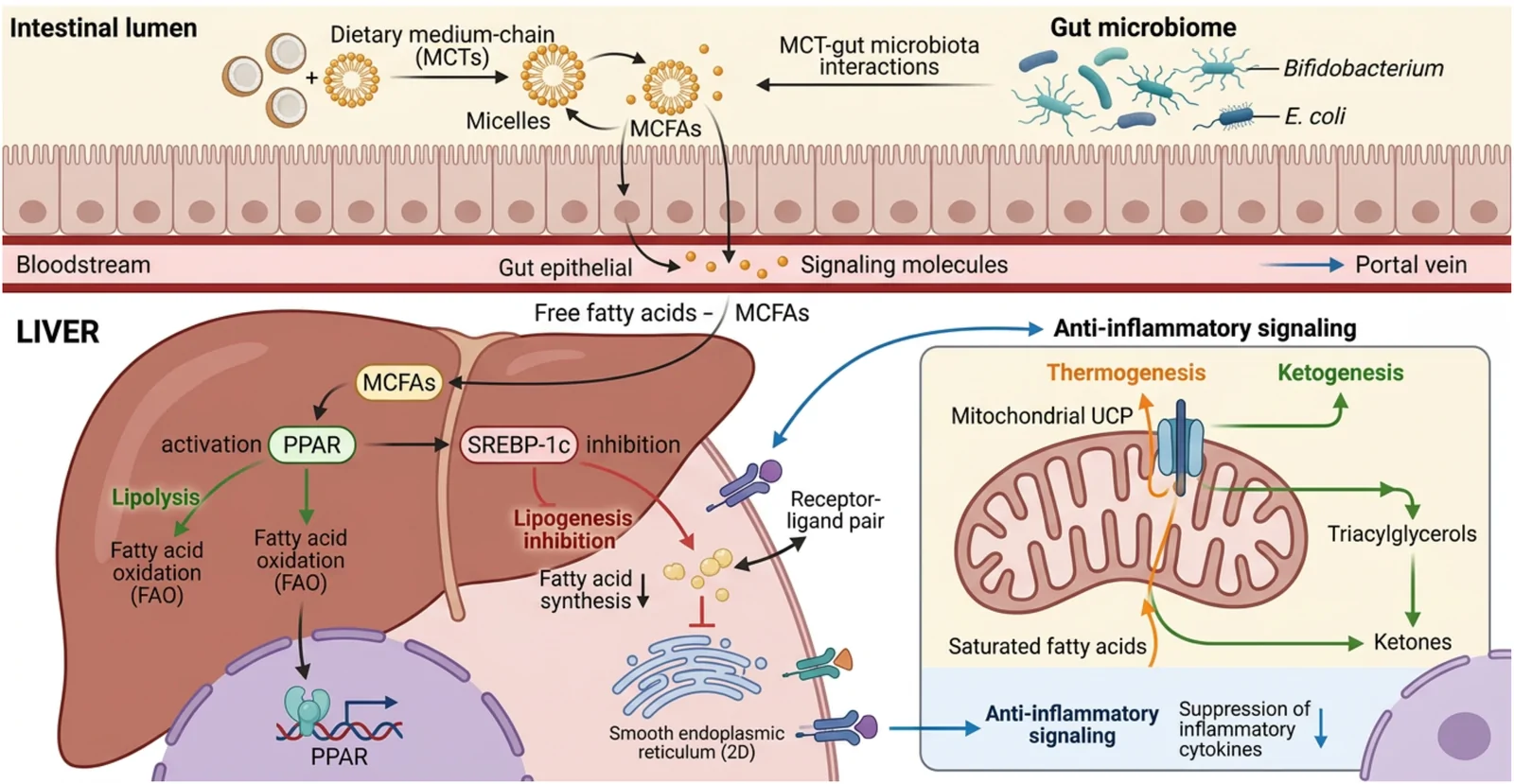
Integrated molecular mechanism network of MCT-mediated lipid metabolic remodeling.

## 4 MCT-mediated lipidomic remodeling: mechanistic and clinical implications for lipid-related diseases

### 4.1 Reprogramming of circulating triglyceride metabolism

MCFAs reach the liver through the portal vein and undergo rapid, largely CPT-1-independent β-oxidation and ketogenesis (Cao et al., 2025; Kanta et al., 2025). Thus, MCFAs provide a smaller pool for re-esterification of TG than do LCFAs and represent a reduced lipid source for the assembly of very-low-density lipoprotein (VLDL) in the liver. MCT supplementation alters the composition of bile acids in the liver in preclinical models of non-alcoholic fatty liver disease (NAFLD) with increased chenodeoxycholic acid (CDCA) intrahepatic concentrations. This bile acid remodeling results in downstream FXR signaling, which transcriptionally represses key lipogenic genes, such as FASN) and ACC, and ultimately leads to reduction of TG stores for VLDL lipidation and packaging (Yue et al., 2024). In parallel, MCFAs inhibit the proteolytic processing of sterol regulatory SREBP-1c, which is the canonical transcriptional regulator of hepatic *de novo* lipogenesis, further limiting endogenous TG anabolism (Jadhav and Annapure, 2023). Hepatic VLDL biogenesis is also regulated by the bioavailability of apolipoprotein B-100 (apoB) and microsomal TG transfer protein (MTP) activity. MCTs have a neutral or inhibitory effect on apoB-dependent VLDL assembly in most dyslipidemic conditions, as opposed to LCTs which promote the secretion of apoB from the liver. Intracellular determinants of VLDL assembly remain under active investigation, and the intracellular tissue plasminogen activator (tPA)-PAI-1 interaction has been shown to govern VLDL assembly in hepatocytes (Dai et al., 2023); this mechanism has not been tested under MCT exposure and is noted here as a hypothesis for future work rather than an established MCT effect. Postprandial lipemia response is the most consistent metabolic response to MCT consumption. A randomized crossover study of 16 individuals with mild-to-moderate hypertriglyceridemia showed that MCFA-containing test meals did not result in an overt increase in plasma TGs, whereas saturated and monounsaturated long-chain lipids did. Thus, postprandial TG incremental area under the curve (iAUC) was consistently decreased after MCT intake, suggesting diet-induced replacement of LCTs with MCTs is an effective nutritional approach to reduce postprandial hypertriglyceridemia in high-dyslipidemic groups (Folwaczny et al., 2021). Chronic MCT supplementation in obese people maintains resting metabolic rate and optimizes hepatic ketogenesis, thus potentially improving peripheral glucose homeostasis and lowering baseline fasting glycemia. However, the effect of MCT on fasting TG level is still debatable in clinical studies. A meta-analysis combining data from qualified randomized controlled trials showed that high dose MCT supplementation resulted in a slight increase (about 0.14 mmol/L) in fasting TG levels compared to LCT supplementation (McKenzie et al., 2021). However, in metabolically healthy adults, moderate MCT consumption over 12 weeks was not found to affect lipid profiles in the circulation, including TG and apoB levels (Hoang et al., 2024). These inconsistent clinical results, therefore, indicate that the lipid-regulating effect of MCT is cohort-specific and dose-dependent in that MCT supplementation is more effective at reducing acute postprandial lipid fluctuations than chronic fasting hypertriglyceridemia.

### 4.2 Differential modulation of cholesterol lipid subfractions

One of the metabolic key points of distinction between purified MCT oil and traditional coconut oil is the differential modulation of cholesterol lipid subfractions. Coconut oil is often touted as a source of MCT, but its content of lauric acid (C12) and myristic acid (C14) invariably results in marked increases in total cholesterol (TC) and LDL-C (Sankararaman and Sferra, 2018). Purified MCT oils have a nonhypercholesterolemic effect because it is known that they are absorbed by the portal circulation and metabolized by hepatic mitochondria (Duranova et al., 2025). Consumption of coconut oil has been found to raise LDL-P, while MCT intake has been shown to diminish the number of small dense LDL (sdLDL) (**Figure 3**) subfractions, which are found to be atherogenic. This remodeling is of great clinical relevance because sdLDL particles have a greater affinity for arterial proteoglycans and are prone to oxidative modification, which are both major factors in development of atherosclerotic plaques (Vekic et al., 2022). Purified MCTs reduce the traditional LDL risk of the long-chain saturated fats in whole coconut products by favoring larger LDL subclasses and having a neutral effect on LDL-P. In addition to LDL remodeling, the lipidomic effects of MCTs are also beneficial for high-density lipoprotein (HDL) functionality and reverse cholesterol transport (RCT). MCT mediated metabolic signaling has been demonstrated to activate important enzymes, including lecithin-cholesterol acyltransferase (LCAT) and phospholipid transfer protein (PLTP) (Lee et al., 2018). These enzymes play a critical role in the maturation of nascent HDL particles and effective clearance of cholesterol from peripheral tissues, back to the liver for excretion in bile (Huang et al., 2021). Coconut oil may raise absolute HDL-C levels, sometimes as a side effect of its lauric acid content, but purified MCT oil specifically enhances the cholesterol efflux capacity of HDL lipids (Duarte et al., 2022). The traditional coconut oil is nearly 50% lauric acid, which is classified as a MCFAs, but acts like a LCFA on hepatic HMG-CoA reductase activity and LDL receptor expression (Mumme and Stonehouse, 2015). Comparative trials showed that the removal of C12 significantly affects the TC: HDL ratio and lowers plasma LDL levels when enzymatic interesterification is used to produce low-lauric acid lipid profiles than when virgin coconut oil is used (Chatturong et al., 2023). This purification process allows metabolically beneficial properties of MCFAs (such as quick energy release and satiety) to be utilized without side effects of inducing an atherogenic lipid profile (Maher et al., 2017).

**Figure 3:**
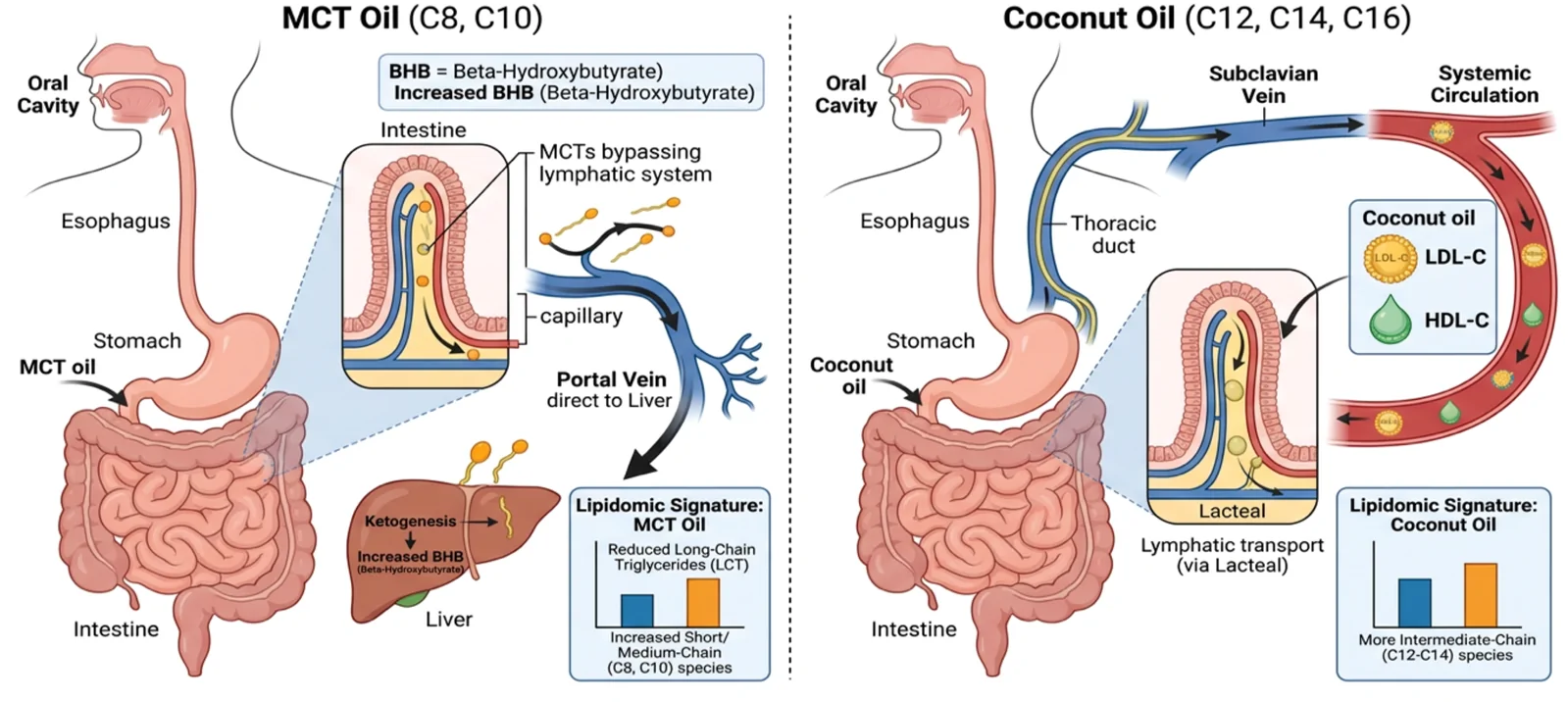
Lipidomic profiling alterations following MCT supplementation compared with coconut oil intervention.

### 4.3 Modulation of residual atherogenic lipid risk biomarkers

Beyond LDL-C, the shift from lipid measurements to a more detailed evaluation of residual cardiovascular risk has raised the possibility that MCTs may have a role in altering other biomarkers of atherogenesis. Currently, non-high-density lipoprotein cholesterol (non-HDL-C) and remnant cholesterol are considered to be better markers of atherosclerotic cardiovascular disease (ASCVD) risk and capture the total burden of all apolipoprotein B (ApoB)-containing lipoproteins (Hansen et al., 2024). MCTs are transported mainly through the portal vein and are rapidly oxidized in the liver, which helps to prevent postprandial increase of remnant-like particles and bypass the exogenous chylomicron pathway. Studies involving specialized populations, including individuals with Familial Chylomicronemia Syndrome, have shown that the replacement of LCTs in the diet with MCTs can effectively decrease serum apoC-III and remnant lipoprotein cholesterol, which results in a reduction of remnant lipoprotein cholesterol (RLC) residual lipid risk (Toth and Banach, 2025). However, in healthy normolipidemic subjects the evidence is less consistent; some prospective studies have shown a lack of effect on non-HDL-C and ApoB levels following 12-week interventions with MCT, suggesting that the regulatory actions of MCTs may depend on the baseline metabolic status and degree of underlying hypertriglyceridemia (Hoang et al., 2024). In particular, the effect of MCT supplementation on the residual risk marker lipoprotein(a) [Lp(a)] has been relatively sparse in the recent literature, with the majority of trials reporting either no effect or only modest reductions in comparison with other TG-lowering agents (Yue et al., 2024). Recent studies using a high throughput lipidomics approach have shown that dietary MCTs can selectively shift the lipidome, including the molecular species of phosphatidylcholines and sphingomyelins, that are linked to plaque stability and inflammatory signaling (Airhart et al., 2016). Comparative studies of the effects of MCTs with common vegetable oils showed that MCTs are more efficient at reducing lipid accumulation in the liver and enhancing systemic lipid profiles through the upregulation of the farnesoid X receptor (FXR) signaling pathway and the modulation of bile acid metabolism (Kanta et al., 2025). This change is important, as the relative contribution of remnant cholesterol to non-HDL-C is now acknowledged to be a key determinant in secondary prevention, with a higher proportion of remnant cholesterol in non-HDL-C being linearly related to the risk of ASCVD. MCTs could present a therapeutic opportunity for better risk stratification in patients who have not been able to reach LDL-C targets despite being at high risk. However, the predictive value of the MCT-modulated lipid profile needs to be interpreted with caution: after fifteen months of MCT supplementation in Alzheimer’s disease patients, plasma eicosapentaenoic acid (EPA) and docosapentaenoic acid (DPA) levels were lowered while HDL-C and LDL-C remained unchanged, introducing new metabolic trade-offs (Juby et al., 2022).

### 4.4 Sources of heterogeneity in lipid response to MCT supplementation

The effect of MCT supplementation on metabolism is quite variable and largely determined by the type of fatty acids and intervention parameters. The ratio of octanoic acid (C8:0) to decanoic acid (C10:0) is one of the most important factors for the ketogenic efficiency, with pure C8:0 oils resulting in a steeper and stronger dose-dependent rise in β-hydroxybutyrate (βHB) levels than C10:0 or mixed blends (Juby et al., 2021). Clinical studies have indicated a linear dose-response relationship between ketogenesis and dose up to ~ 42g, but even modest ketogenic doses (2–6g/d) have shown marked physiological response (e.g. increase in postprandial fat oxidation, body composition changes over 3-month periods) (Ezaki, 2024). The duration of intervention on the other hand is important as long-term effects on traditional lipid markers such as LDL-C and HDL-C are more variable. For example, the benefits of MCT supplementation on traditional lipid markers have been less pronounced in 12-week trials when supplemented as an adjunct to a normal diet without caloric restrictions, indicating that the duration of supplementation should be carefully considered in combination with total energy balance and MCT formulation (Hoang et al., 2024).

In addition to the formulation, host phenotypic modifiers have a significant influence on the individual lipidomic response. Baseline adiposity, such as BMI and Visceral Fat Area (VFA), has been identified as one of the main factors to predict metabolic sensitivity to MCTs. People with higher adiposity (BMI 25–30) tend to have improved fat oxidation and energy expenditure after MCT consumption, but may have lower absolute amounts of ketones produced than lean individuals because of differences in insulin sensitivity and endogenous fatty acid mobilization (Nosaka et al., 2022). A further source of heterogeneity is age: low dose MCT blends have been seen to have beneficial effects on reducing sarcopenia and increasing muscle-to-fat ratios in older and frail individuals, but not in metabolically flexible younger populations. The metabolic utilization of MCTs is also suggested to be age-dependent, as seen in clinical situations from preterm, where special lipid emulsions are needed, to elderly, where neurodegenerative diseases are a clinical concern, possibly due to differences in mitochondrial efficiency and basal metabolic rate throughout the life span (Jadallah and Hammad, 2024). The clinical effects of MCT supplementation are also modified by the baseline level of lipids in the person’s body and their typical diet. MCTs provide a clear benefit over LCTs in patients with mild to moderate hypertriglyceridemia because MCTs do not cause a significant postprandial TG rise, which reduces the risk of postprandial lipemia. The people with preexisting metabolic comorbidities (dyslipidemia and simple obesity) show significantly greater decreases in blood TGs and greater improvement in HOMA-IR when switching from LCTs to pure MCTs than do healthy normolipidemic individuals (He et al., 2024). In addition, the response size is determined by the ratio of long-chain saturated fatty acids (LCSFAs) to unsaturated fats in the habitual dietary lipid matrix. However, meta-analyses indicate that replacing LCSFAs with MCFAs can significantly increase the levels of HDL-C and apolipoprotein A-I, highlighting the importance of diet as a filter for lipid remodeling achieved by MCTs (Panth et al., 2018). Finally, the effectiveness of MCTs depends on the balance between the C8/C10 ratio of the oil, the dosage and the general metabolic health and dietary habits of the person taking them (Hoang et al., 2024). Summary of MCT vs. CCO effects on circulating lipid biomarkers is summarized in (**Table 2**).

**Table 2:** Summary of MCT vs. CCO effects on circulating lipid biomarkers.

Biomarker	Healthy subjects	Prediabetes	Type 2 diabetes	Hypertriglyceridemia	Atherosclerosis risk	Reference
TG	↓	↓	↓	↓↓	↓	(Panth et al., 2018)
TC	↔	↓	↔	↓	↔	(Palazhy et al., 2014)
LDL-C	↔	↓	↓	↔	↓	(Woollett et al., 1989)
Small dense LDL	↔	↔	↓	↓	↔	(Palazhy et al., 2014)
HDL-C	↑	↔	↔	↔	↔	(da Silva Lima and Block, 2019)
Remnant-C	↓	↓	↓	↓	↓	(Wang et al., 1996)
Lp(a)	↔	↔	↔	↔	↔	(Tholstrup et al., 2004)

Symbols: ↑ increase/↓ decrease/↔ no significant change relative to CCO.

## 5 Translational clinical lipid evidence and heterogeneity resolution in health and lipid disorders

The clinical translation of MCT research has historically been clouded by the conflation of pure MCT oils with CCO. Recent meta-synthesized evidence from randomized controlled trials (RCTs) has started to address this heterogeneity, especially in the context of body composition and regulation of adipose lipids (McKenzie et al., 2021).

### 5.1 Body composition and adipose lipid regulation

Meta-analyses of RCT lipid evidence have provided a solid basis for MCT oil’s efficacy in visceral adipose lipid reduction and systemic body weight management. The meta-analysis studies showed that the use of diets enriched with pure MCTs resulted in a statistically significant but modest weight reduction, corresponding to a weighted mean difference of -1.62% compared to LCT intervention; the effect size is small, and its clinical interest lies in its consistency across trials rather than in its magnitude (He et al., 2024). The portal-oxidative route helps create a negative energy balance by increasing postprandial energy expenditure and diet-induced thermogenesis (Mumme and Stonehouse, 2015). Medium to long chain TGs (MLCT) have been shown to cause substantial reductions in waist circumference and hip circumference in overweight and obese subjects following a 60-day intervention (Nosaka et al., 2023). One key feature of the metabolically healthy lipid phenotype is preferential loss of white adipose lipid mass, accompanied by maintenance of lean tissue mass. MCTs may exert a nitrogen-sparing effect. MCT supplementation (6 g/day) for 3 months led to a significant increase in muscle mass and muscle grip strength and a decrease in fat mass in a frail older population (Ezaki, 2024). This lean tissue preservation is probably due to the rapid oxidation of MCTs that are used as an alternative energy source to glucose by the skeletal muscle, thus protecting against muscle protein breakdown during caloric deficit (Harvey et al., 2018). Moreover, MCTs affect satiety by slowing the emptying of the stomach, and stimulating satiety hormones such as peptide YY and leptin, which, by inhibiting compensatory overeating, are key to preserving the lean-to-fat mass ratio, as has been observed in conventional weight-loss programs (Maher et al., 2019). Coconut oil is commonly touted as containing MCTs, but it is mainly composed of lauric acid (C12:0) which is more of an LCT in terms of transport and storage. The meta-analysis study showed that no significant difference between consumption of coconut oil and other plant oils in terms of body weight (MD -0.24 kg) or waist circumference (MD -0.64 cm) with a total of 515 participants. Conversely, pure MCT oil interventions have been shown to have a more consistent anthropometric benefit. The implications for the lipid profile are also quite different: coconut oil raises HDL-C by about 3.28–7.91 mg/dL, whereas C8/C10 MCT oil has a more dramatic effect on lowering TGs and promoting weight loss (Duarte et al., 2022).

### 5.2 Glycemic-lipid homeostasis and insulin sensitization in dyslipidemic populations

The effectiveness of MCT oil is strongly associated with the metabolic condition of the individual before taking MCT oil, ranging from healthy normolipidemic adults to prediabetic dyslipidemia and/or type 2 diabetic hyperlipidemia. However, in metabolically flexible adults without diabetes, a modest increase in MCT oil intake of 30g/day over 6 weeks resulted in increased insulin sensitivity, though not significant for fasting glucose or HOMA-IR, indicating that in these individuals, the homeostatic buffer is sufficient to cope with moderate lipid modulation (Thomas et al., 2019). In addition, acute doses of pure C8 or C10 MCTs of the magnitude used in ketogenic-response studies, typically 10–30 g given as a single bolus, do not significantly affect glucose levels in healthy cohorts in the absence of exogenous carbohydrate, although co-ingestion with 50 g of glucose may slightly modify the insulinogenic response (Heidt et al., 2023). By comparison, the therapeutic effect of MCTs is significantly greater in populations with metabolic dysfunction. Systemic reviews and meta-analyses of clinical trials consistently show that diets composed of pure MCTs produce better metabolic outcomes than LCT diets, particularly in individuals with overweight, obesity, and/or prediabetic dyslipidemia. These are reflected in an improvement in HOMA-IR scores and in the stabilization of fasting glucose, which represents a normalization of insulin signaling pathways (He et al., 2024). The supporting mechanisms derive principally from animal and cell-based work rather than from short-term human trials. In mice, dietary decanoate (C10:0) improved glucose tolerance and conferred resistance to high-fat-diet-induced obesity through GPR84-mediated GLP-1 secretion from enteroendocrine cells (Nonaka et al., 2022), and a high-fat diet enriched in C8:0/C10:0 MCTs triggered hepatic thermogenesis and improved metabolic health in lean and obese mice (Rial et al., 2020). In humans, substituting a small proportion of dietary LCFAs with MCFAs protected against insulin resistance during short-term caloric excess (Lundsgaard et al., 2021), whereas 14 days of C8:0 MCT oil in healthy adults did not alter markers of NLRP3 inflammasome activation (Neudorf et al., 2022), indicating that an anti-inflammatory contribution has not yet been demonstrated in metabolically healthy humans. The switch to lipid support based on MCT provides double benefits in type 2 diabetic hyperlipidemic groups where parallel changes in lipid profile and glycemic control are achieved at the same time. Quantitative data showed that replacing traditional dietary fats with MCT oil resulted in a substantial decrease in HbA1c and reduction in postprandial hyperglycemia, possibly due to the rapid oxidation of the MCFAs in the mitochondria that spare glucose utilization and enhance metabolic flexibility (Sung et al., 2018). In particular, clinical trials using diabetic models have shown that circulating TGs, LDL-C and non-esterified fatty acids are decreased, while the HDL-) and the HDL-C/LDL-C ratio are increased (Sung et al., 2018). Dosage, however, requires caution in clinical use, since the use of MCTs has been shown to cause hepatotoxic effects in certain rodent models, such as transient hepatic lipid accumulation and focal hepatic inflammation, at intakes corresponding to approximately 20-30% of total energy as MCT or roughly 1 g/kg body weight per day and above, effects that are amplified by concurrent fructose feeding; these intakes substantially exceed the 15–30 g/day typically used clinically in adults (Chamma et al., 2017; Guimarães et al., 2019).

### 5.3 Cardiometabolic lipid risk reduction and vascular lipid protection

The portal absorption allows for more rapid mitochondrial β-oxidation, which avoids peripheral deposition of fat and insulin-mediated lipogenesis, the processes associated with longer-chain fats (Cao et al., 2025). MCTs have a multitude of effects on the vascular lipid environment with normalization of TG and cholesterol profiles; the rapid oxidation of MCFAs prevents hepatic and intramuscular lipid accumulation, and thus reduces the substrate for VLDL production (Titov et al., 2019). MCTs can result in greater decreases in TGs compared to LCT-rich diets and favorable modulation of HDL cholesterol levels. This substrate partitioning, favoring thermogenesis and ketone production over visceral fat storage, mitigates the systemic lipotoxicity that often precedes vascular damage (Bhavsar and St-Onge, 2016). Moreover, MCTs have been shown to offset several of the adverse cardiovascular effects normally seen with long-chain SFAs, specifically the elevation of LDL-C and apoB-containing particles, endothelial dysfunction with impaired flow-mediated dilatation, increased vascular inflammatory signaling and exaggerated postprandial lipemia, indicating that the metabolic effect of SFAs is highly carbon-chain dependent (Shi et al., 2025). In addition to lipid normalization, MCTs are bioactive signaling molecules that strengthen the endothelial lipid barrier, of which caprylic acid (C8:0) has proven to be a potent modulator of vascular inflammation. In atherosclerotic models, C8:0 has been shown to inhibit the activation of the Toll-like receptor 4 (TLR4)/nuclear factor-kappa B (NF-κB) signaling pathway. MCTs can effectively inhibit the expression of mRNA of inhibitory-kappa B kinases, IKKα and IKKβ, which in turn effectively attenuates the release of pro-inflammatory cytokines such as tumor necrosis factor alpha (TNF-α) and interleukin-6 (IL-6) (Zhang et al., 2019). This signaling inhibition prevents inflammatory activation of macrophages in the arterial wall, a pivotal step in the development of atherosclerotic lesions, and consequently stabilizes the endothelial lipid barrier by dampening of oxidative stress and the integrity of tight junctions, thus reducing the lipid-induced endothelial damage and atherogenesis in the vascular wall (Zhang et al., 2019). Large cohort studies, including the Italian adult study (n=1,936), have recently shown that higher chronic consumption of MCFAs is associated with lower diabetes and dyslipidemia and may improve long-term cardiometabolic risk markers (Currenti et al., 2022). Experimental studies in ApoE deficient models also have shown that atherosclerotic lesions in the aortic sinus and aorta area are significantly decreased after long-term supplementation with MCT (Zhang et al., 2019). While additional large-scale cohort studies with purified MCT intervention are still needed to confirm the current conclusions, existing preclinical and clinical evidence suggests that MCT oil is a protective functional lipid that has vascular-protective effects, mediated through metabolic optimization and anti-inflammatory signaling without an increase in the long-term risk of ASCVD (Cao et al., 2025).

### 5.4 Therapeutic lipid applications in ketogenic diet therapy and neuro-lipid metabolic support

MCTs are easily hydrolyzed and transported via the portal vein directly to the liver where they are oxidized as obligatory substrates for ketone body production, namely β-hydroxybutyrate (βHB) and acetoacetate. This metabolic pathway enables a state of nutritional ketosis (blood βHB levels of 0.5-2.0 mmol/L) without the severe low carb intake seen with classic ketogenic diets (Newport, 2023). A 6-month trial of patients with mild cognitive impairment (MCI) has confirmed that the daily consumption of about 30 g of ketogenic MCTs is safe and well-tolerated, with no harmful effects on circulating levels of cardiometabolic or inflammatory markers compared with calorie-matched placebos (Myette-Côté et al., 2021). This heterogenicity resolution of the lipid response indicates that the use of MCT as a component of a therapeutic strategy does not necessarily worsen cardiovascular risk factors during ketosis (Giudici, 2021). MCT oil is also a powerful cerebral lipid fuel supplement that helps to offset the brain-energy gap associated with glucose hypometabolism in aging and in neurodegenerative diseases like Alzheimer’s disease (AD) (Croteau et al., 2018). Under these conditions, the potential for the brain to metabolize glucose may be reduced by 20% to 40%, with a relatively spared capacity to metabolize ketone bodies (Espina et al., 2022). Brain ketone uptake has been confirmed by PET imaging to be directly proportional to plasma ketone concentrations after MCT use, effectively doubling the available energy substrate in AD patients (Croteau et al., 2018). MCT-derived products produce ketones increasing acetyl-CoA level and mitochondrial efficiency in the cerebral cortex. Additionally, the cognitive effects seem to depend on genetic background, with some studies finding that cognitive improvements are greater in patients without the APOE4 gene (Xu et al., 2020). In addition to energetics, MCTs cause neuro-protective effects via anti-inflammatory signaling, and may reduce beta-amyloid and tau pathology, thus providing a multi-faceted therapeutic approach for neuro-lipid metabolic support (Rebello et al., 2015).

### 5.5 Critical reconciliation of conflicting clinical lipid data

The metabolic effect of MCT oil on lipid profiles has been the subject of much academic debate, with a range of results from beneficial cardioprotective effects to neutral and even detrimental effects on hypercholesterolemia. These discrepancies must be reconciled, and a detailed consideration of methodological differences, including dosing schemes and the metabolic status of the populations, is required (McKenzie et al., 2021). Lower doses (such as less than 15–20 g/day) might be better tolerated, but are unlikely to reach the level necessary to produce significant changes in hepatic fatty acid oxidation or TG clearance (He et al., 2024). On the other hand, higher doses (up to 30 g/day) are metabolically more active, but are often constrained by adverse gastrointestinal effects, resulting from low adherence and reduced quality of data in long-term studies (Chapman-Lopez and Koh, 2022). Another factor is the duration of the intervention; meta-analysis indicates a stronger reduction in lipids in shorter interventions (8 weeks or less) with sources such as virgin coconut oil (VCO) (Zhang et al., 2025). These early lipid improvements can be dampened by compensatory physiological mechanisms or reduced diet adherence over longer periods of time, resulting in the null findings of multi-month longitudinal studies. It is important to recognize that MCTs can cause relative increases in LDL-C when compared to unsaturated fatty acids, but have a neutral or even positive lipid profile when compared to LCSFAs. The isolated effect of MCTs is complicated by the fact that it is hard to distinguish the effect when the background diet is not strictly controlled for total fatty acid composition. Another feature of protocol heterogeneity is the separation of pure MCTs and MLCT mixtures or coconut oil products. In overweight and obese people, use of pure MCTs seems to aid in the reduction of TGs and the improvement of HOMA-IR more effectively than MLCTs (He et al., 2024). The effectiveness of the MCT supplementation seems to be state-dependent as those with metabolic disorders or obesity showed greater decreases in TG levels after supplementation than healthy normal weight people (Zhang et al., 2025). On the other hand, normal weight adults (BMI < 25 kg/m2) have been found to have paradoxical effects in terms of lipid profile, with production of higher levels of total and LDL cholesterol, especially when MCTs are included when following a very-low-carbohydrate ketogenic diet (Joo et al., 2023). Therefore, the clinical interpretation of the data needs to be separated from a *“one size fits all”* approach, emphasizing the importance of the interplay of the length of the MCT chains, their dosage and the metabolic status of the target group. Key clinical randomized controlled trials investigating MCT Oil supplementation for lipid and metabolic outcomes are summarized in (**Table 3**).

**Table 3:** Key clinical randomized controlled trials investigating MCT Oil supplementation for lipid and metabolic outcomes.

Disease status	Study population	MCT formulation & daily dosage	Intervention length	Primary lipid/Metabolic endpoints	Key outcomes	Confounding limitations	Reference
Healthy	Healthy adults undergoing supervised ketogenic-diet induction (n=28)	100% MCT oil (C8:0/C10:0); 30 mL, three times/day vs sunflower oil (LCT) control	20 days; randomized parallel-group	Serum β-hydroxybutyrate (βHB), fasting glucose	MCT supplementation produced higher βHB concentrations at all measured time points and accelerated nutritional ketosis (although not statistically significant). Increased gastrointestinal symptoms, particularly abdominal discomfort, were reported.	Small sample size; short intervention period; lipid profile parameters (TC, LDL-C, HDL-C, TG) were not primary outcomes; healthy young population limits applicability to metabolic disease groups.	(Harvey et al., 2018)
Healthy	Healthy volunteers receiving MCT capsules as an intended neutral placebo comparator	MCT oil capsules (C8:0/C10:0); crossover design	4 weeks per intervention arm; double-blind placebo-controlled crossover	HDL-C, LDL-C, C-reactive protein	MCT supplementation unexpectedly altered HDL-C, LDL-C, and inflammatory markers, indicating that MCT oil may not represent a metabolically inert placebo.	Lack of detailed washout information; study originally designed for placebo validation rather than evaluating MCT efficacy.	(Ferguson et al., 2016)
Prediabetes/Insulin Resistance Risk	Overweight non-diabetic adults with insulin resistance risk (n=22)	MCT oil replacing part of dietary fat; 30 g/day per 2000 kcal intake	6 weeks	Fasting insulin, HOMA-IR, adiponectin, fasting ketones	No significant improvements were observed in fasting glucose, insulin, HOMA-IR, or insulin sensitivity index. Adiponectin levels decreased significantly.	Single-arm feasibility study without control group; causal interpretation is limited.	(Thomas et al., 2019)
Prediabetes/Insulin Resistance Risk	Healthy lean men exposed to short-term hypercaloric high-fat diet as an insulin resistance model (n=17)	Saturated medium-chain fatty acids (MCFA), ~30 g/day (5% energy), substituted for long-chain fatty acids (LCFA)	3 days; randomized parallel-group	Hyperinsulinemic-euglycemic clamp-derived insulin sensitivity, plasma glucose, ketones	MCFA substitution attenuated diet-induced insulin resistance compared with LCFA supplementation during short-term overfeeding.	Extremely short intervention; experimental model rather than clinically diagnosed prediabetes; small sample size.	(Lundsgaard et al., 2021)
Type 2 Diabetes Mellitus (T2D)	Moderately overweight, free-living T2D patients from China (n=40)	MCT oil; 18 g/day incorporated into meals vs corn oil (LCT) control	90 days; randomized parallel-group	Total cholesterol, triglycerides, HOMA-IR	MCT supplementation reduced body weight, waist circumference, HOMA-IR, and total cholesterol compared with baseline and LCT control. Serum C-peptide levels increased.	Free-living design with self-reported dietary compliance; modest sample size; limited population diversity.	(Han et al., 2007)
Hyperlipidemia	Chinese adults with hypertriglyceridemia (n=112)	Medium- and long-chain triglyceride (MLCT) oil; 25–30 g/day as primary cooking oil vs LCT oil	8 weeks; double-blind randomized controlled trial	Serum triglycerides, LDL-C, body fat percentage	MLCT oil produced greater reductions in body fat, triglycerides, and LDL-C compared with LCT oil.	Cooking oil intervention limits precise dosage control; improvements may partly reflect changes in body weight and total fat intake.	(Zhang et al., 2009)
Hyperlipidemia	Hypertriacylglycerolemic overweight Chinese adults stratified by BMI (n=101)	MLCT oil; 25–30 g/day as cooking oil vs LCT control	8 weeks; double-blind randomized controlled trial	Serum TAG, LDL-C, visceral and subcutaneous fat area	Improvements in TAG and LDL-C were dependent on BMI, with the strongest effects observed in participants with BMI 24–28 kg/m².	*Post hoc* BMI subgroup analysis reduces statistical power; single ethnic cohort; cooking-oil format affects dose precision.	(Zhang et al., 2010)
Neurological/Ketogenic Therapy	Patients with mild-to-moderate Alzheimer’s disease (n=152)	AC-1202 (caprylic acid/C8 MCT emulsion; Ketasyn) administered as part of normal diet	90 days; randomized, double-blind, placebo-controlled multicenter trial	Serum βHB elevation; lipid profile monitored as safety outcome	MCT intervention rapidly increased circulating ketones and improved ADAS-Cog cognitive scores, particularly in APOE4-negative and treatment-compliant participants.	Lipid outcomes were secondary; cognitive effects influenced by APOE4 genotype; industry-sponsored study.	(Henderson et al., 2009)
Neurological/Ketogenic Therapy	Mild-to-moderate Alzheimer’s disease patients in crossover trial with open-label extension	MCT oil titrated up to 42 g/day	15 months (double-blind crossover plus open-label extension)	TC, LDL-C, HDL-C, TG, body composition, glucose metabolism	Long-term MCT supplementation demonstrated safety with no adverse effects on lipid profile, body composition, or glucose metabolism.	Small sample size; broad variation in age and disease severity; gastrointestinal intolerance limited achievement of target dosage in some participants.	(Juby et al., 2022)

## 6 MCT lipid safety profiling, lipid-related risks and evidence limitations

### 6.1 Dose-dependent gastrointestinal lipid intolerance and tolerance thresholds

Acute gastrointestinal (GI) intolerance is often a limiting factor for the clinical use of MCT oil as a metabolic therapeutic. Because MCTs are absorbed rapidly by the portal route, an acute high dose influx may over tax the mucosal absorptive capacity. This gives rise to a dose-limiting toxicity that manifests a spectrum of symptoms such as epigastric pain, bloating, nausea, flatulence and osmotic diarrhea (Lin et al., 2021). These side effects can be severe in children, especially when receiving treatment for refractory epilepsy, which can entail behavioral food refusal, requiring careful management of the lipid delivery protocol (Yu et al., 2022). Recent studies have confirmed that the ketogenic response, defined as an increase in blood βHB levels, has a relatively linear dose-response up to about 42g, whereas the threshold for GI tolerance is highly idiosyncratic with a strong non-linear relationship (Juby et al., 2021). Some people will experience acute discomfort if they consume more than 10-15g of pure MCT oil at a single meal. Most current clinical guidelines recommend using a conservative dose of 5g (about one teaspoon) and gradually increasing to a therapeutic target of 15-20g of specific fatty acids, including octanoic acid (C8) (Lin et al., 2021). A key limitation is the absence of uniformized tolerance provocation tests, which results in considerable heterogeneity in reporting and quantifying GI distress in clinical trials. The individual tolerance levels, as measured by research on healthy younger adults, older adults and AD patients, do not depend significantly on age, sex or sarcopenic status (Meer and Fischer, 2024). Reported rates of GI symptoms vary widely between trials and populations rather than converging on a single population estimate, and no validated prevalence figure for non-tolerance in the general population is currently available; individual phenotypes, e.g., BMI and visceral fat distribution, may play a more important role in modulating the metabolic output, apart from the primary GI symptomology (Juby et al., 2021). It indicates that there is inter-individual variability, which means that a *“one size fits all”* dosage recommendation would not be suitable for clinical application. The best method will be the physical alteration of the lipid delivery system, with emulsified preparations of MCT oils showing consistently improved GI tolerability. This is probably because of the greater surface area for enzyme activity and slower rate of gastric emptying. In addition, recent studies in children with epilepsy have demonstrated a reduction of GI side effects from more than 60% to about 30%, or even less, for a mixed-lipid diet consisting of a 1:1 ratio of MCTs to LCTs, without a decrease in the therapeutic effect (Yu et al., 2022; Han et al., 2021). Finally, timing of the ingestion is of great importance, as taking MCTs with a low carbohydrate meal reduces the osmotic load and makes them much safer to ingest during the induction period of supplementation (Lin et al., 2021).

### 6.2 Systemic lipid metabolic risks of excessive MCT lipid exposure

MCTs have garnered a great deal of attention for their rapid oxidation and ketogenic properties in regards to their therapeutic use. But the systemic lipid metabolic risks of unregulated high dose exposure is a big concern. MCTs have been shown to raise blood ketone body levels, including BHB, for neuroprotective effects, however, there is pharmacokinetic evidence of a linear, but highly variable, dose-response relationship (Juby et al., 2021). A typical 42 g dose of MCT oil can raise βHB levels from a mild 0.4 mM to a greater 2.1 mM, depending on the person’s basal BMI, visceral fat level, and previous diet. The observed inter-individual variability is also noteworthy, indicating that it is possible to alter the metabolic state of individuals to non-pathological pathways of ketosis with high dose supplementation, which may affect metabolic signaling pathways or acid-base balance without clinical supervision (Juby et al., 2021). In addition, the perception of MCTs as solely fat burning lipids is contradicted by the report of hepatic lipid overload in chronic or high doses. High level of MCTs has been shown to induce severe hepatic abnormalities such as the induction of hepatic steatosis and focal liver inflammation (Guimarães et al., 2019). This biochemical overload is due to the unanticipated induction of *de novo* lipogenesis, along with concurrent changes in mitochondrial beta-oxidation pathways. In mice fed isocaloric diets with matched total fat intake, an MCT-rich diet increased rather than decreased hepatic fat accumulation, contrary to the reduction predicted by the oxidative profile of MCTs; this has been attributed to concurrent stimulation of hepatic lipogenic markers with suppression of β-oxidation markers (Chamma et al., 2017). This metabolic burden is enhanced by additional diets stressors such as fructose, which together with the MCTs, synergistically effects hepatic damage and lipogenic flux. A number of specific ketogenic protocols recommend prolonged administration of MCT food and the use of MCTs for extended durations could pose a risk of hepatic lipid sequestration, and lipid processing dysfunction, based on the evidence from general high-dose supplementation (Fukazawa et al., 2021). Another major drawback to excessive consumption of MCTs is that a caloric surplus can result in unwanted adiposity. For instance, the primary byproduct of tricaprylin (C8 MCT) metabolism, octanoic acid, has been identified as a ligand for PPAR-γ (Li et al., 2023). This pathway is activated to stimulate the adipogenic differentiation of preadipocytes and to upregulate genes that promote fat storage, thereby increasing the size of the adipocytes and the total amount of fat tissue. This apparently contradicts the adipose *PPAR-γ* benefit and the ROS-mediated *PPAR-γ* inactivation described. The three observations are reconcilable if *PPAR-γ* signaling is treated as context-dependent rather than uniformly beneficial or harmful: under isocaloric substitution at moderate doses, MCFA-associated PPAR-γ activity in adipose tissue favors adiponectin secretion, beige remodeling and improved insulin sensitivity (Watanabe and Tsujino, 2022; Ye et al., 2019); in isolated adipocytes, octanoate generates reactive oxygen species that inactivate *PPAR-γ* and suppress lipogenic gene expression (Guo et al., 2006); and under caloric surplus with sustained high-dose exposure, ligand-driven activation predominates and favors adipogenesis (Li et al., 2023). Energy balance, dose and model system, rather than any single directional effect, therefore determine the net outcome. Also, the effect of MCTs in appetite regulation is bi-directional: they can decrease hypothalamic leptin resistance, but ingested MCFAs are also used directly for the n-octanoyl acyl modification of ghrelin, increasing gastric acylated ghrelin without altering total ghrelin content (Nishi et al., 2005). Activation of this orexigenic pathway may offset the suppression of food intake by ketones, thereby adding to a chronic excess of calories.

### 6.3 Systematic limitations of current lipid evidence base

The clinical translation of MCT oil research is currently hindered by several structural deficits within the available evidence base, most notably the lack of long-term prospective trials. Although short-term intervention (4–12 weeks) has shown successful acute metabolic effects of the use of MCTs, including increased thermogenesis and rapid ketogenesis, there has been a considerable lack of research focused on chronic atherosclerotic lipid endpoints (Heidt et al., 2025). Surrogate markers (including circulatory TC, LDL-C, and TGs) have been extensively used without focusing on clinical endpoints, such as major adverse cardiovascular events (MACE) or the progression of carotid intima-media thickness. Therefore, long-term effects of a continued intake of MCT on the longitudinal progression of atherosclerosis are only speculative and backed by only weak human clinical studies (Li et al., 2015). There is a general lack of a universal and standardized MCT lipid formulation, and validated lipid-modulating dosage guidelines is a secondary systematic limitation. Much of the research does not differentiate between pure MCT oils typically containing C8 and C10 oils, and MLCTs that have different pharmacokinetic and functional properties (He et al., 2024). Moreover, dosages are still rather arbitrary and studies have used doses from 15g daily to as high as 1g/kg of body weight. Lastly, there is a lack of lipid safety and efficacy data in vulnerable dyslipidemic patient populations. There are mechanistic studies in animal models and healthy human volunteers that show favorable or neutral results with regard to lipids, but this cannot be extrapolated with any degree of safety to those with pre-existing metabolic diseases (Margata et al., 2018). Patients with conditions like lipoprotein lipase deficiency (LPLD), familial hypercholesterolemia or severe metabolic syndrome are generally not included in the mainstream MCT trials because of the possible confounding factors (Aljouda et al., 2023). Therefore, the lack of clinical clarity regarding the possibility that MCTs could worsen the lipid overload in patients with impaired lipid clearance warrants discussion about the risks and benefits of employing MCTs in clinical practice. However, systematic reviews have repeatedly indicated that there is a need for appropriately powered, randomized controlled trials in these dyslipidemic groups to determine if MCT formula actually provides a true safety benefit over the use of conventional LCT formulas (He et al., 2024). Therefore, the clinical application of MCT therapy in cardiovascular medicine should be prudently limited until the group of high-risk population is thoroughly investigated (Muscaritoli and Pradelli, 2021).

## 7 Mechanism-lipid phenotype causality, lipid evidence reconciliation and translational lipid therapeutic implication

Purified MCT oil is a metabolically unique lipid compared to CCO. The CCO to its lauric acid rich and LCFAs substituted structure, has consistently been demonstrated to raise TC, LDL-C, and HDL-C compared to non-tropical vegetable oils (Neelakantan et al., 2020). In pooled randomized trial data from only MCT oil preparations with >98% purity of C6:0-C10:0, there were no significant effects on TC, LDL-C, or HDL-C; TG concentration was only slightly and control-dependently elevated (McKenzie et al., 2021). Comparative meta-analyses also report that the atherogenic lipid profile traditionally associated with coconut oil is mainly due to the LCFAs (lauric, myristic, and palmitic) and not to C8:0/C10:0 fatty acids (Panth et al., 2018; Duarte et al., 2022). Therefore, the reclassification of purified MCT oil as an independent lipid modulator, not as a stand-in for coconut oil, is the conceptual and empirical correction that is needed for this, and it has implications for how MCFAs is interpreted by practitioners, regulators and guideline committees. The C8:0/C10:0 fatty acids appear to bypass chylomicron assembly and enter the portal circulation directly, which allows for a fast oxidative disposal and ketogenesis independent of the activity of CPT-1 at the hepatocellular and adipocyte level (Cao et al., 2025). In adipocytes, octanoate has been reported to inhibit lipogenic gene expression by reactive oxygen species (ROS) mediated inactivation of *PPAR-γ* which provides a likely explanation for its adiposity-lowering but LDL-neutral effects in clinical cohorts (Guo et al., 2006);, this effect is dose- and energy balance-dependent and coexists with ligand-mediated *PPAR-γ* activation under caloric surplus. This mechanistic concept is supported by human postprandial data, which showed that an MCFA based test meal induced a near zero excursion of chylomicron-TG, and reduced the increased response of chylomicron-TG after long chain saturated fat or monounsaturated fat consumption in mildly hypertriglyceridemic individuals directly connecting a measurable and clinically relevant lipid phenotype to the proposed mechanism of substrate-bypass (Folwaczny et al., 2021). The consistency of the null results across different exposure settings, from a 90-day trial in free-living type 2 diabetic adults (Han et al., 2007) to a 12-week ApoB-focused trial in healthy adults (Hoang et al., 2024) and to pooled short-duration RCTs (McKenzie et al., 2021), would be unlikely if there were significant residual confounding. Despite this uniformity at the aggregate level, there is clinical response heterogeneity that is a significant challenge for translation. One is the presence of genotype dependent responsiveness in Alzheimer’s disease trials, with cognitive benefit and changes in metabolomics being limited to those who were not APOE4 positive (Xu et al., 2020). Second, the co-administered fat matrix seems to influence outcome, e.g., the combination of MCT oil with saturated butter fat (versus MCT oil alone) led to more favorable ApoB and LDL-C trends (Hoang et al., 2024). Third, unexpected changes in background lipid levels have been reported; after fifteen months of MCT supplementation in Alzheimer’s disease patients, plasma eicosapentaenoic acid (EPA) and docosapentaenoic acid (DPA) levels were lowered, whereas HDL-C and LDL-C levels remained unchanged, an effect that is not yet understood. None of these subgroup specific effects can be detected by pooled mean difference meta-analyses suggesting that null aggregate results might mask clinically interesting heterogeneity in specific subgroups (Juby et al., 2022). Three considerations must therefore be weighed together. The effect of purified MCT oil on the lipid profile is generally favorable to neutral. Against this sit a small but reproducible increase in TG and the newly identified risk of essential fatty acid displacement when dosed over longer periods. In favor are the metabolic benefits of moderate weight loss, reduced waist circumference and improved glycemic parameters in overweight patients with type 2 diabetes (He et al., 2024). For the majority of healthy and metabolically at-risk adults, this trade-off supports cautious and monitored supplementation; however, in populations with pre-existing borderline omega-3 status or hypertriglyceridemia, more stringent lipid-panel surveillance is warranted (Juby et al., 2022). Translationally, these results also highlight the need to move beyond general MCFAs recommendations in clinical lipid management guidelines and to embrace a precision-nutrition approach in which the dose of MCT oil, the co-administered fat matrix, treatment duration, and genotype (such as APOE status) are explicitly modeled as effect modifiers, rather than variance to be accounted for in the usual way (Mukarromah et al., 2023). Thus, use of purified C8:0/C10:0 MCT oil as opposed to whole coconut oil as an ingredient in functional lipid-therapeutic foods is a more scientifically sound basis for cardiometabolic and neurological adjunct therapy, provided that future trials are randomized controlled studies with additional pre-specified genotype- or phenotype-based subgroups analyses and routine essential fatty acid monitoring (Nosaka et al., 2022).

## 8 Future strategic lipid research outlook

Though the mechanistic and short- to medium-term clinical data shown favorable result but the field is still limited by the lack of large, long-term, randomized controlled trials that would be able to determine whether the lipid neutral-to-favorable phenotype of purified MCT oil can be replicated in terms of reduction of atherosclerotic cardiovascular events or all-cause mortality (McKenzie et al., 2021). There are no meta-analyses available for interventions longer than 12 week and the longest published neurological trial enrolled only twenty participants over 15 months (Juby et al., 2022). Hence, adequately powered, multi-year randomized controlled trials with hard atherosclerotic endpoints and mortality tracking, standardized MCT formulations and pre-specified apolipoprotein B and non-HDL-cholesterol tracking are an urgent need, especially considering the unexpected essential fatty acid-lowering signal reported with prolonged exposure (Juby et al., 2022). The second priority is to create a precision lipid-nutrition profile for MCT oil. Currently, the ratios of C8:0 to C10:0, the type of fat matrix and dosage used daily vary significantly, from approximately 6 g/day in frail older adults to roughly 1 g/kg body weight in clinical nutrition practice (Ezaki, 2024; Li et al., 2015) and there is no agreement on the best fat matrix (i.e. formulation) and dosage (C8:0 to C10:0) to use for different metabolic phenotypes. Future dose-ranging and formulation-comparison trials, ideally with factorial designs to separate out the effect of C8:0 from that of C10:0, are needed to establish minimum effective and maximum tolerable doses in healthy, dyslipidemic, and neurodegenerative populations, as well as to determine if genotypic markers like apolipoprotein E4 status significantly stratify response to treatment as preliminary evidence indicates (Xu et al., 2020; Henderson et al., 2009). Preclinical data supports the restoration of gut microbial diversity and partial reversal of gut dysbiosis by the use of MCTs and classical KDs, without maintaining a chronic state of ketosis (M’Bra et al., 2025; Majumdar et al., 2023), and shows that the brain metabolic regulation of C8:0 differs from C10:0, via the astrocyte-neuron lactate-shuttle pathway (Thevenet et al., 2016). A longitudinal study that combined fecal metagenomics, hepatic and cerebral lipidomics and the circulating kinetics of the ketone bodies would clarify whether the lipid and cognitive phenotypes observed in humans are mainly caused by hepatic lipid reprogramming, metabolites produced by the microbiome, or direct central fatty acid signaling, and would help explain why others, such as APOE4-positive subjects, do not benefit (Xu et al., 2020). Applying this evidence to the general healthy adult population, the current data support the careful addition of purified MCT oil, as metabolically neutral with respect to TC, LDL-C, and HDL-C, and permit the use of MCT oil up to approximately 30 g/day in divided doses, with a note of caution about the slight, but reproducible, TG-raising effect, which should be monitored by periodic testing of lipid panels instead of being used freely. MCT oil is not recommended as an alternative lipid-lowering treatment in dyslipidemic and high CVD risk populations, especially in patients with pre-existing hypertriglyceridemia, but may be used as an adjunctive dietary alternative fat source for LCFAs as it has been shown to reduce postprandial TG elevation compared with the long-chain saturated and long-chain monounsaturated fat (Folwaczny et al., 2021). Baseline and follow-up lipid panels, inclusive of non-HDL-cholesterol and, where feasible, apolipoprotein B, are advisable in this group before and during supplementation (Meer and Fischer, 2024). In clinical auxiliary settings, such as ketogenic therapy for epilepsy, mild cognitive impairment and Alzheimer’s disease, MCT oil supplementation should be tailored according to APOE genotype when available, with routine monitoring of essential PUFA status such as EPA and DPA, which is depleted by prolonged administration, and should not be used as a metabolically inert placebo comparator in future trial design (Juby et al., 2022; Ferguson et al., 2016). The use of purified, standardized C8:0/C10:0 MCT oil, not CCO, as the main ingredient in a functional lipid-therapeutic food or clinical nutrition product should be the basis across all three tiers, pending long term outcome data confirming.

## 9 Conclusion

The nutritional paradigm of medium-chain triglycerides (MCTs) has shifted broadly from routine dietary supplementation to precise metabolic modulation, with this review establishing that purified MCT oil concentrated in caprylic (C_8_:0) and capric (C_10_:0) acids delivers distinct lipid-regulating mechanisms absent in conventional coconut oil abundant in lauric acid (C_12_:0). Unlike CCO, which travels via lymphatic circulation and risks chylomicron-driven ectopic lipid accumulation, C_8_/C_10_ MCTs enter the portal vein directly to undergo rapid hepatic oxidation and thermogenesis, bypassing canonical lipid storage pathways to drive clinically validated benefits such as weight loss in obese subjects and alleviated hepatic fat deposition in NAFLD. Beyond substrate metabolism, purified MCTs coordinate core signaling networks including the AMPK-UCP1 thermogenic axis and FXR-dependent bile acid homeostasis, mechanistically explaining suppressed pro-atherogenic lipid levels and improved insulin sensitivity. Heterogeneous interindividual metabolic responses, however, require patient stratification by baseline metabolic and enzymatic biomarkers to standardize therapeutic efficacy, reconciling contradictory prior study outcomes and advancing precision nutrition frameworks. Clear differentiation between pharmaceutical-grade C_8_/C_10_ MCTs and unrefined coconut oil is therefore indispensable for contemporary lipid management; upcoming clinical guidelines and functional therapeutic food development should prioritize uniform C_8_/C_10_ lipid profiles to unlock their translational value for metabolic and cardiovascular protection, allowing clinicians to deploy purified MCTs as a validated nutritional intervention to restore systemic metabolic balance and curb lipid-mediated chronic illnesses.
